# Dual regulation of β_2_-adrenoceptor messenger RNA expression in human lung fibroblasts by β_2_–cAMP signaling; delayed upregulated inhibitors oppose a rapid in onset, direct stimulation of gene expression

**DOI:** 10.1007/s00210-014-0971-7

**Published:** 2014-04-08

**Authors:** N. Kämpfer, F. Lamyel, I. Schütz, M. Warnken, K. Hoffmann, I. von Kügelgen, Kurt Racké

**Affiliations:** Institute of Pharmacology & Toxicology, University of Bonn, Biomedical Center, Sigmund-Freud-Str. 25, D-53105 Bonn, Germany

**Keywords:** β_2_-Adrenoceptor expression, Adenylyl cyclase, Lung fibroblasts, PKA, Epac

## Abstract

Based on their bronchodilatory effect, β_2_-adrenoceptor agonists constitute essential elements in the treatment of bronchial asthma and COPD. As treatment with β_2_-adrenoceptor agonists has been associated with worsening of airway hyper-reactivity, possibly because of loss of β-adrenoceptor function, molecular mechanism of the regulation of β_2_-adrenoceptor expression were studied. MRC-5 human lung fibroblasts were cultured in absence or presence of test substances followed by β_2_-adrenoceptor messenger RNA (mRNA) determination by qPCR. After inhibition of mRNA synthesis by actinomycin D, β_2_-adrenoceptor mRNA decreased with a half-life of 23 min, whereas inhibition of protein synthesis by cycloheximide caused an about 5- and 6-fold increase within 1.5 and 4 h, respectively. β_2_-Adrenoceptor mRNA was increased by about 100 % after 1 h exposure to formoterol or olodaterol but decreased by about 60 % after 4 h agonist exposure. Both effects of β_2_-adrenoceptor agonists were mimicked by forskolin, a direct activator of adenylyl cyclase and cholera toxin, which stimulates adenylyl cyclase by permanent activation of Gs. β_2_-Adrenoceptor agonist-induced upregulation of β_2_-adrenoceptor mRNA was blocked by the β_2_-adrenoceptor antagonist ICI 118551 and prevented by actinomycin D, but not by cycloheximide. Moreover, in presence of cycloheximide, β_2_-adrenoceptor agonist-induced reduction in β_2_-adrenoceptor mRNA was converted into stimulation, resulting in a more than 10-fold increase. In conclusion, expression of β_2_-adrenoceptors in human lung fibroblasts is highly regulated at transcriptional level. The β_2_-adrenoceptor gene is under strong inhibitory control of short-living suppressor proteins. β_2_-Adrenoceptor activation induces via adenylyl cyclase - cyclic adenosine monophosphate (cAMP) signaling a rapid in onset direct stimulation of the β_2_-adrenoceptor gene transcription, an effect opposed by a delayed upregulation of inhibitory factors.

## Introduction

Based on their bronchodilatory effect, β_2_-adrenergic agonists constitute an essential element in the treatment of bronchial asthma and COPD (e.g., Sin et al. 2003; Barnes 2004; Walters et al. 2005; Fitzgerald and Fox 2007; Cazzola et al. 2011). However, there is increasing evidence that they may exert a number of additional effects of potential therapeutic value. Thus, we recently showed that human lung fibroblasts express β_2_-adrenoceptors which mediate various inhibitory effects on pro-fibrotic features (Lamyel et al. 2011). On the other hand, treatment with long-acting β_2_-adrenoceptor agonists has been associated with possible worsening of airway hyper-reactivity (e.g., Martinez 2005; Nelson 2006; Cockcroft 2006; Cazzola et al. 2011), possibly because of loss of β_2_-adrenoceptor function. There is evidence for a complex agonist-mediated modulation of β_2_-adrenoceptor responsiveness, involving effects effect at the transcriptional and post-transcriptional levels. A large number of studies documented downregulation of β_2_-adrenoceptor density after prolonged agonist exposure, which involves several mechanisms, including downregulation of β_2_-adrenoceptor messenger RNA (mRNA) (e.g., Bouvier et al. 1989; Hadcock et al. 1989; Collins et al. 1989; Hosoda et al. 1995; Tittelbach et al. 1998). On the other hand, there is evidence that β-adrenoceptor agonist exposure can via cAMP-signaling enhance β_2_-adrenoceptor gene expression (Collins et al. 1989). However, substantial cell specific differences in the regulation of β_2_-adrenoceptor mRNA appear to be exist (Danner and Lohse 1997).

Therefore, the present study aimed to analyze molecular mechanisms involved in the regulation of β_2_-adrenoceptor expression in human lung fibroblasts, which have been identified as a new target for drugs used in the treatment of chronic obstructive airway diseases (Racké et al. 2008). In particular, a potential time-dependent modulation of β_2_-adrenoceptor mRNA expression by β_2_-adrenoceptor activation and the down-stream cAMP signaling pathway were explored.

Preliminary reports of some of the data have been given (Warnken-Uhlich et al. 2011, Kämpfer et al. 2012).

## Materials and methods

### Culture of lung fibroblasts

MCR-5 human lung fibroblasts (CCL-171, ATCC, Manassas, USA) were grown in Eagle’s MEM supplemented with 10 % FCS, 2 mM L-glutamine; Earle’s BBS adjusted to contain 2.2 g/l sodium bicarbonate, 0.1 mM non-essential amino acids, 1.0 mM sodium pyruvate, 100 U/ml penicillin, and 100 μg/ml streptomycin. Cells were grown in a humidified incubator at 37 °C and 5 % CO_2_ and passaged by trypsinization at nearly confluence.

### Extraction of RNA and real-time reverse transcription-polymerase chain reaction

Total RNA was isolated by help of silica-gel-based membranes according to manufacturer’s instructions including an additional DNase digestion protocol to beware any contamination by genomic DNA (Qiagen, Hilden, Germany). First strand cDNA was synthesized using Omniscript reverse transcriptase (Qiagen).

Quantitative PCR was performed by monitoring the fluorescence of SYBR Green dye on a Statagene Mx3000P real-time PCR system. Applied primer pairs (based on human EMBL sequences) were specific for the β_2_-adrenoceptor 5′-GATTTCAGGATTGCCTTCCAG-3′ and 5′GTGATATCCACTCTGCTCCCC-3′ and the housekeeping gene GAPDH, 5′-CTGCACCACCAACTGCTTAGC-3′ and 5′-GGCATGGACTGTGGTCATGAG-3′ which were used for normalization. The cycling conditions were the following: 10 min polymerase activation at 95 °C and 40 cycles at 95 °C for 30 s, 59 °C for 30 s, and 72 °C for 30 s. The threshold was automatically set by the software. The crossing point of the amplification curve with the threshold represents the “Ct.”

Fluorescence data from each sample were analyzed with the 2^−[∆∆Ct]^ method: fold induction = 2^−[∆∆Ct]^, where ∆∆Ct = [Ct GI (unknown sample) − Ct GAPDH (unknown sample)] − [Ct GI (calibrator sample) − Ct GAPDH (calibrator sample)], GI is the gene of interest.

### Analysis of cellular cyclic AMP accumulation

Cellular cAMP levels were determined as described previously (Hoffmann et al., 2008). In brief, MRC-5 cells were cultured on 24-well plates for 24 h. After removal of the culture medium, cells were incubated with HBSS buffer at 36.5 °C for 2 h followed by additional 10 min, 60 min, or 4 h in absence or presence of test substances or solvent control (DMSO). The reaction was stopped by removal of the reaction buffer followed by the addition of a hot lysis solution (Na_2_EDTA 4 mM, Triton X 100 0.01 %, pH 7.5). cAMP levels in the supernatant were then quantified by incubation of an aliquot with cAMP-binding protein and [^3^H] cAMP (Perkin Elmer, Boston, USA), and liquid scintillation counting after removal of the unbound cAMP by charcoal. cAMP levels per well were calculated by regression analysis from a standard curve determined for each experiment. cAMP levels were expressed either in absolute values (pmol/500 μl) or as percent of the mean value observed in presence of forskolin in each cell preparation.

### Statistical analysis

All values are means with SEM of *n* experiments. Statistical significance of differences was evaluated by ANOVA followed by Dunnett or Bonferroni test using GraphPad InStat (GraphPad Software, San Diego, USA). *P* < 0.05 was accepted as significant.

### Drugs and materials

Formoterol was a gift from AstraZeneca (Lund, Sweden) and olodaterol from Boehringer Ingelheim (Biberach, Germany). All other drugs were purchased: actinomycin D, cholera toxin, cycloheximide, forskolin, isoprenaline, IBMX (2-isobutyl-1-methylxanthine), orciprenaline, penicillin-streptomycin solution, and trypsin from Sigma (Deisenhofen, Germany); ICI 118,551 ((±)-1-[(2,3-dihydro-7-methyl-1H-inden-4-yl)oxy]-3-[(1-methylethyl) amino]-2-butanol hydrochloride) from Biozol (Eching, Germany); 6-Bnz-cAMP (N^6^-benzyladenosine-3′,5′-phosphate) and 8-pCPT-2′–O-Me-cAMP (8-(4-chlorophenylthio)-2′–O-methyladenosine-cAMP) from Biolog Life Science Institute (Bremen, Germany); desoxynucleotide mixture from Fermentas (St. Leon-Rot, Germany); Eagle’s minimal essential medium (MEM) with Earl’s salts and L-glutamine, non-essential amino acids from PAA (Cölbe, Germany); fetal calf serum (FCS) from Biochrom (Berlin, Germany); Taq DNA-polymerase from Invitrogen (Karlsruhe, Germany); and Omniscript reverse transcriptase, RNeasy Mini kit, QuantiTect^TM^ SYBR Green PCR kit, and RNase-free DNase set from Qiagen (Hilden, Germany). Oligodesoxynucleotides for qPCR were obtained from Eurofins MWG Operon (Ebersberg, Germany).

## Results

**Fig. 1:**
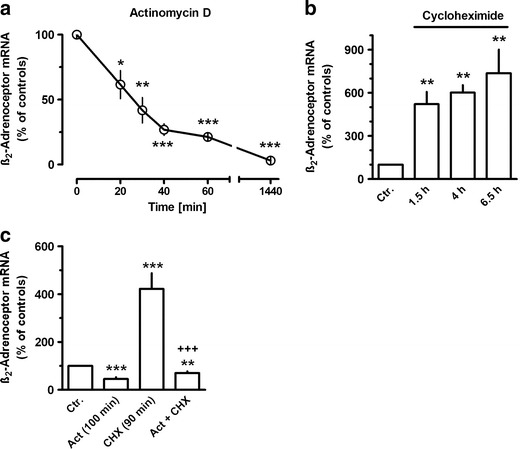
Time-dependent effects of actinomycin D (*Act*, 30 μM) and/or cycloheximide (*CHX*, 30 μM) on β_2_-adrenoceptor mRNA expression in MRC-5 human lung fibroblasts. After dissemination, cells were cultured for 24 h in presence of 10 % FCS followed by up to 24 h in FCS-free medium in absence or presence of test drugs. When Act and CHX were present together (**c**), Act was present 10 min before the addition of CHX for further 90 min. Thereafter, total RNA was isolated, treated with DNase and used for quantitative real-time PCR. Ordinate (**a**) and height of columns (**b**, **c**): β_2_-adrenoceptor mRNA (−2^ΔΔCt^ × 100) is expressed as percent of the respective control of the individual cell preparation, given are means with SEM of *n* ≥ 5. Significance of differences: *P < 0.05; **P < 0.01; ***P < 0.001 vs respective control; ^+++^ *P* < 0.01 vs CHX

By using quantitative real-time PCR, the present study confirms previous observations based on semi-quantitative RT-PCR that human lung fibroblasts express significant amounts of mRNA encoding β_2_-adrenoceptors. Under control conditions, the β_2_-adrenoceptors mRNA levels, expressed as ΔCt over GAPDH, amounted to 12.1 ± 0.1 (*n* = 60) and were very similar in several series of experiments. After inhibition of de novo RNA synthesis by actinomycin D, β_2_-adrenoceptor mRNA showed a rapid decline, with a half-life of about 23 min (Fig. 1a). On the other hand, inhibition of protein synthesis by cycloheximide resulted in rapid, marked increase in β_2_-adrenoceptor mRNA, about 5-fold within 1.5 h and only slightly higher after 4 and 6 h (Fig. 1b). Actinomycin, present 10 min prior to cycloheximide, almost prevented the increase induced by cycloheximide (Fig. 1c).

**Fig. 2:**
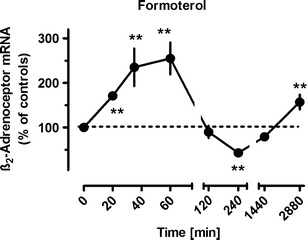
Time-dependent effects of formoterol on β_2_-adrenoceptor mRNA expression in MRC-5 human lung fibroblasts. After dissemination, cells were cultured for 24 h in presence of 10 % FCS followed by up to 48 h in FCS-free medium in absence or presence of formoterol (100 nM). Thereafter, total RNA was isolated, treated with DNase and used for quantitative real-time PCR. *Ordinate*: β_2_-adrenoceptor mRNA (−2^ΔΔCt^ × 100) is expressed as percent of the respective control of the individual cell preparation, given are means ± S.E.M. of *n* ≥ 6. Significance of differences: **P < 0.01 vs respective control

**Fig. 3:**
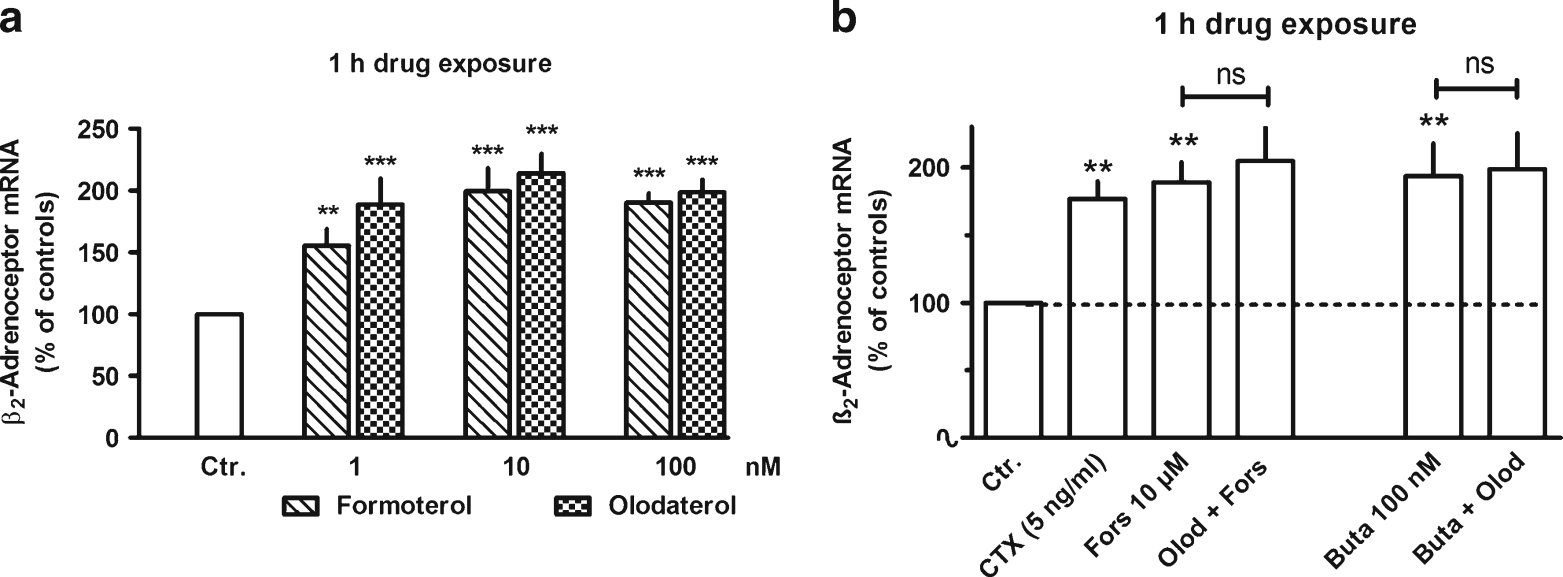
Comparison of the concentration-dependent effects of formoterol and olodaterol (**a**) and the effects of cholera toxin (*CTX*), forskolin (*Fors*), butaprost (*Buta*), and olodaterol (*Olod*, 10 nM) (**b**) on β_2_-adrenoceptor mRNA expression in MRC-5 human lung fibroblasts. After dissemination, cells were cultured for 24 h in presence of 10 % FCS followed by 1 h in FCS-free medium in absence or presence of test drugs at the concentrations given. Thereafter, total RNA was isolated, treated with DNase and used for quantitative real-time PCR. *Height of columns*: β_2_-adrenoceptor mRNA (−2^ΔΔCt^ × 100) is expressed as percent of the respective control of the individual cell preparation, given are means + SEM of *n* ≥ 6. Significance of differences: **P < 0.01; ***P < 0.001 vs respective control; not significant (*ns*) vs respective value in absence of Olod

**Fig. 4:**
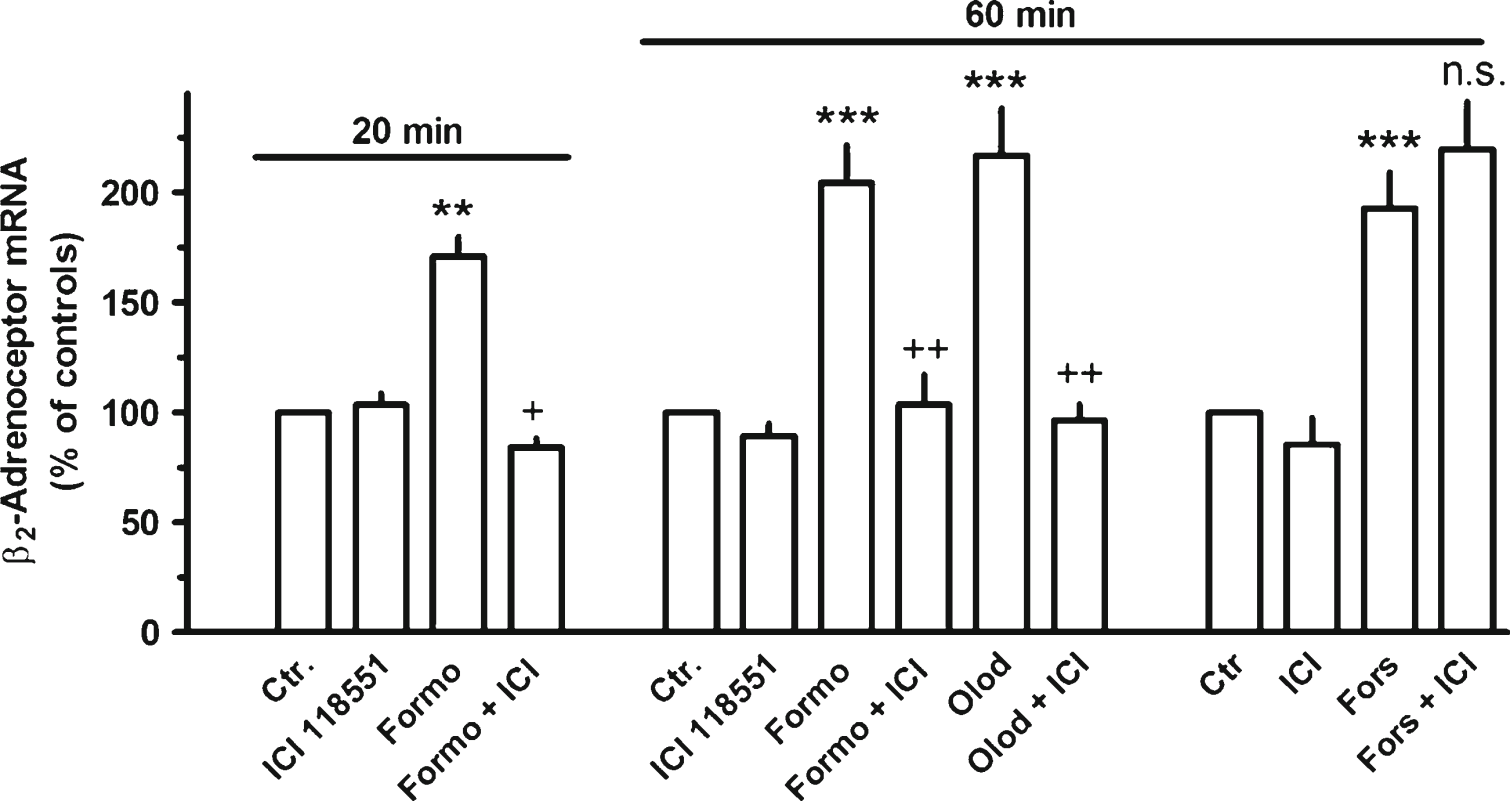
Effects formoterol (*Formo*, 100 nM), olodaterol (*Olod*, 10 nM), forskolin (*Fors*, 10 μM), and/or ICI 118551 (*ICI*, 3 μM) on β_2_-adrenoceptor mRNA expression in MRC-5 human lung fibroblasts. After dissemination, cells were cultured for 24 h in presence of 10 % FCS followed by 20 or 60 min in FCS-free medium in absence or presence of test drugs at the concentrations given. Thereafter, total RNA was isolated, treated with DNase, and used for quantitative real-time PCR. *Height of columns*: β_2_-adrenoceptor mRNA (−2^ΔΔCt^ × 100) is expressed as percent of the respective control of the individual cell preparation, given are means + SEM of *n* ≥ 4. Significance of differences: **P < 0.01; ***P < 0.001 vs respective control; ^+^ *P* < 0.05; ^++^ *P* < 0.01 vs respective value in absence of ICI 118551; not significantly (*ns*) different vs respective value in absence of ICI 118551

**Fig. 5:**
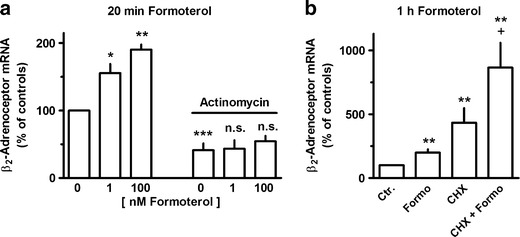
Effects of formoterol (**a** at the concentration given; **b** 100 nM) and/or actinomycin (30 μM) (**a**, **c**) or cycloheximide (CHX, 30 μM) on β_2_-adrenoceptor mRNA expression in MRC-5 human lung fibroblasts. After dissemination, cells were cultured for 24 h in presence of 10 % FCS followed by 1 h in FCS-free medium in absence or presence of test drugs at the concentrations given, actinomycin D being present 10 min and CHX 30 min before formoterol. Thereafter, total RNA was isolated, treated with DNase and used for quantitative real-time PCR. *Height of columns*: β_2_-adrenoceptor mRNA (−2^ΔΔCt^ × 100) is expressed as percent of the respective control of the individual cell preparation, given are means + SEM of *n* ≥ 6. Significance of differences: *P < 0.05; **P < 0.01; ***P < 0.001 vs respective control; ^+^ *P* < 0.05 vs CHX alone; not significant (*ns*) vs actinomycin alone

**Fig. 6:**
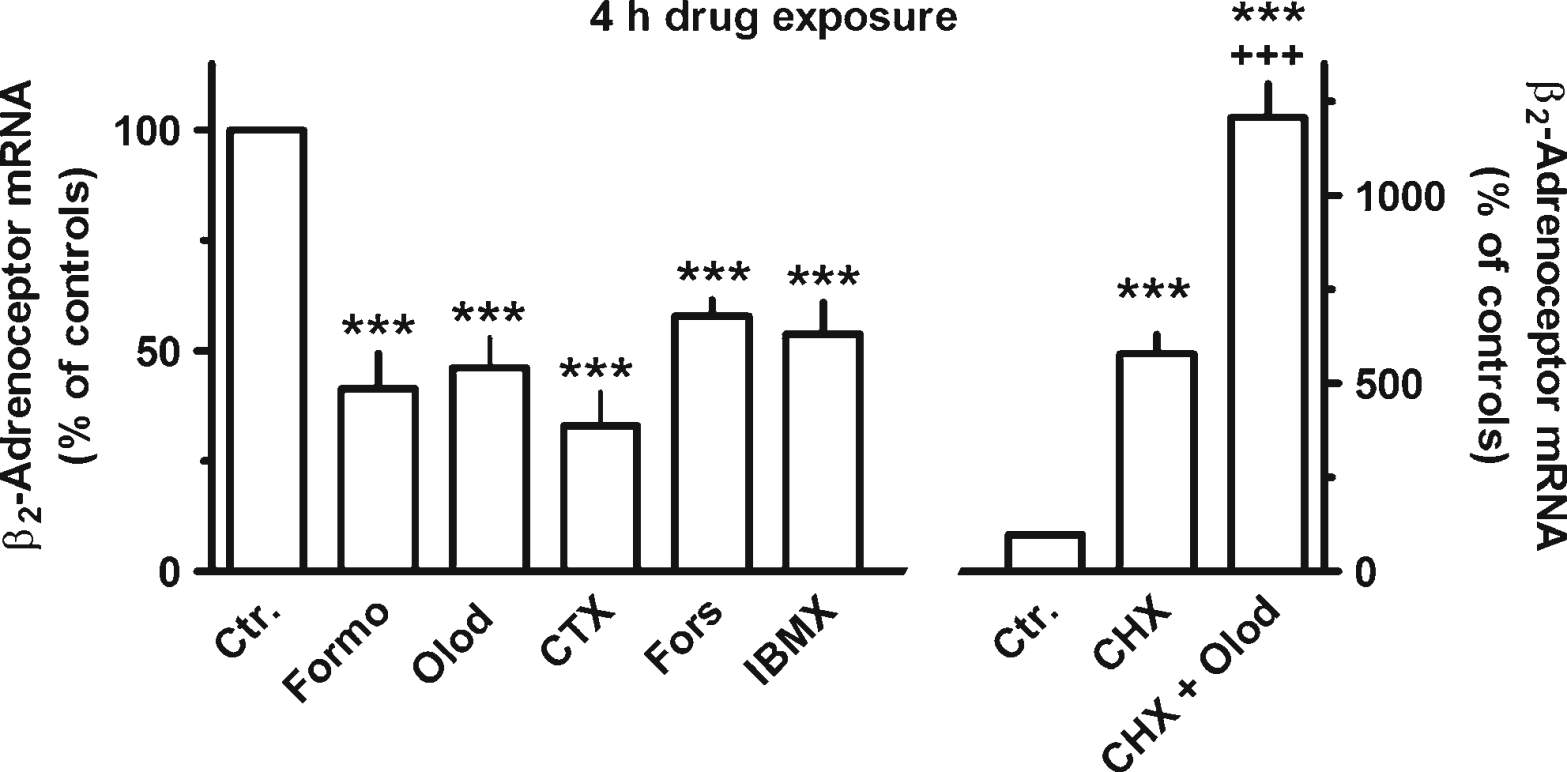
Effects of formoterol (*Formo*, 10 nM), olodatereol (*Olod*, 10 nM), cholera toxin (*CTX*, 5 ng/ml), forskolin (*Fors, 10 µM*) or IBMX (100 μM), ICI 118551 (*ICI*, 3 μM) alone or in combination with Formo (*left hand scale*), or cycloheximide (*CHX*, 30 μM) alone or in combination with Olod (10 nM, *right hand scale*) on β_2_-adrenoceptor mRNA expression in MRC-5 human lung fibroblasts. After dissemination, cells were cultured for 24 h in presence of 10 % FCS followed by 4 h in FCS-free medium in absence or presence of test drugs at the concentrations given, CHX being present 30 min before Olod. Thereafter, total RNA was isolated, treated with DNase and used for quantitative real-time PCR. *Height of columns*: β_2_-adrenoceptor mRNA (−2^ΔΔCt^ * 100) is expressed as percent of the respective control of the individual cell preparation, given are means + SEM of *n* ≥ 6. Significance of differences: ***P < 0.001 vs respective control; ^+++^ *P* < 0.01 vs CHX alone; ^##^ *P* < 0.01 vs Formo alone

Exposure to β_2_-adrenoceptor agonists showed time-dependent opposing effects on β_2_-adrenoceptor mRNA expression. As shown in Fig. 2 for formoterol, β_2_-adrenoceptor agonist exposure resulted in a very rapid increase in β_2_-adrenoceptor mRNA, significantly already after 20 min, and a maximal increase by about 150 % was observed within 1 h. This effect vanished after 2 h, and an inhibition by about 55 % was seen after 4 h, but also this effect was lost over time and an enhanced expression was again seen after 48 h (Fig. 2). Similar effects were also evoked by olodaterol, another long-acting β_2_-adrenoceptor agonist (Bouyssou et al. 2010) (Figs. 3a and 6) as well as by the short-acting agonists isoprenaline and orciprenaline (data not shown). The stimulatory effects of the β_2_-adrenoceptor agonists were mimicked by direct activation of adenylyl cyclase, either by exposure to cholera toxin or to forskolin (Fig. 3b) and also by the prostanoid (EP_2_) receptor agonist butaprost. The effects of forskolin and butaprost were not additive to that of the β_2_-adrenoceptor agonist olodaterol (Fig. 3b). Finally, the selective β_2_-adrenoceptor antagonist ICI 118551 (Baker 2005) prevented the stimulatory (Fig. 4) as well as the inhibitory (Fig. 6) effects of the β_2_-adrenoceptor agonists but did not affect the upregulation caused by forskolin (Fig. 4). After inhibition of de novo RNA synthesis by actinomycin D, the stimulatory effect of formoterol was abolished, whereas in presence of cycloheximide, which by its own caused already a marked increase in β_2_-adrenoceptor mRNA, formoterol elicited a further marked increase (Fig. 5), resulting in an almost 9-fold increase when cycloheximide and the β_2_-adrenoceptor agonist were concomitantly present. The half-life of the β_2_-adrenoceptor mRNA in presence of 100 nM formoterol was about 26 min, i.e., it was not significantly affected by agonist exposure.

Cholera toxin, like forskolin, also mimicked the inhibitory effect of the β_2_-adrenoceptor agonists on β_2_-adrenoceptor receptor mRNA seen after a 4-h drug exposure (Fig. 6). Finally, the 4-h exposure to the phosphodiesterase inhibitor IBMX also caused a reduction in β_2_-adrenoceptor receptor mRNA by about 50 % (Fig. 6). However, it should be mentioned, that short-time exposure (1 h) to IBMX (1–100 μM) did not significantly affect β_2_-adrenoceptor receptor mRNA expression (data not shown). As already described above (Fig. 1), inhibition of protein de novo synthesis by cycloheximide resulted in marked increases in β_2_-adrenoceptor mRNA levels. In presence of cycloheximide, the inhibitory effect of olodaterol (4 h) was prevented and converted into a marked stimulatory effect. Concomitant presence of cycloheximide and the β_2_-adrenoceptor agonist resulted in an about 14-fold increase in β_2_-adrenoceptor receptor mRNA levels (Fig. 6).

**Fig. 7:**
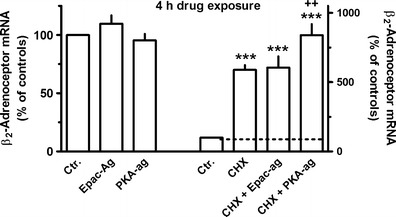
Effects of the selective PKA (6-Bnz-cAMP, 500 μM) or Epac (8-CPT-2′–O-Me-cAMP, 100 μM) agonist (*left hand scale*) or cycloheximide (*CHX*, 30 μM) alone and in combination with the Epac or PKA agonists (*right hand scale*) on β_2_-adrenoceptor mRNA expression in MRC-5 human lung fibroblasts. After dissemination, cells were cultured for 24 h in presence of 10 % FCS followed by 4 h in FCS-free medium in absence or presence of test drugs at the concentrations given, CHX being present 30 min before the Epac and PKA agonists. Thereafter, total RNA was isolated, treated with DNase and used for quantitative real-time PCR. *Height of columns*: β_2_-adrenoceptor mRNA (−2^ΔΔCt^ × 100) is expressed as percent of the respective control of the individual cell preparation, given are means + SEM of *n* ≥ 6. Significance of differences: ***P < 0.001 vs respective control; ^++^ *P* < 0.01 vs CHX alone

Exposure to the selective PKA agonist 6-Bnz-cAMP or the selective Epac agonist 8-CPT-2′–O-Me-cAMP for 1 h (data not shown) or 4 h (Fig. 7) did not significantly affect β_2_-adrenoceptor mRNA levels. However, in presence of cycloheximide, 6-Bnz-cAMP, but not 8-CPT-2′–O-Me-cAMP, caused a significant further increase in β_2_-adrenoceptor mRNA levels at 4 h (Fig. 7).

**Fig. 8:**
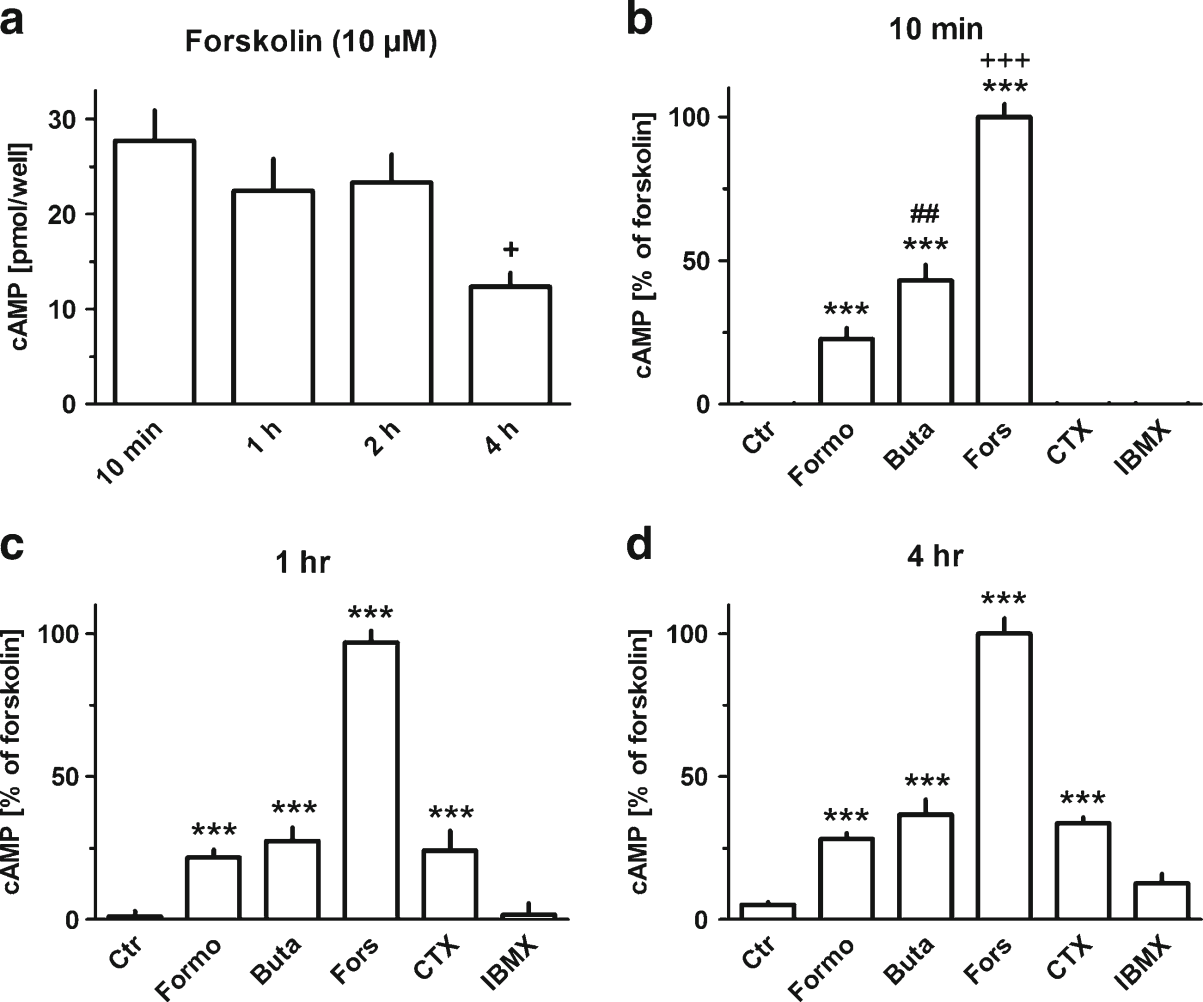
Effects of forskolin (*Fors*, 10 μM), formoterol (*Formo*, 100 nM), butaprost (*Buta*, 100 nM), cholera toxin (*CTX*, 5 ng/ml), or IBMX (100 μM) on cellular cAMP in MRC-5 human lung fibroblasts. After dissemination, cells were cultured for 24 h in presence of 10 % FCS an then for by 2 h in HBSS followed by additional 10 min to 4 h (as indicated) in absence or presence of test drugs. Thereafter, cells were lysed and cAMP levels determined. *Height of columns:* **a** cAMP in picomole/well, **b**-**d** cAMP expressed as percent of the mean levels in presence of forskolin determined in the respective cell preparation, given are means + SEM of *n* ≥ 8. Significance of differences: ***P < 0.001 vs respective control; ^+++^ *P* < 0.01 vs resp. Formo and Buta; ^+^ *P* < 0.05 vs forskolin 10 min, ^##^ *P*<0.01 vs resp. Formo

In further experiments, time-dependent effects of formoterol, butaprost, forskolin, choleratoxin, and IBMX on cellular cAMP were studied. In most experiments, basal cAMP levels were below or very close to the detection limit. Formoterol, butaprost, and forskolin induced a clear increase in cellular cAMP already within 10 min (Fig. 8b). Forskolin caused the strongest increase and cellular cAMP remained at the same level for up to 2 h. After 4 h presence of forskolin, cellular cAMP was still clearly elevated but significantly lower compared to the initial 10 min period (Fig. 8a). In the initial 10 min, the effect of forskolin was compared to that of formoterol about 4-fold and compared to butaprost about 2-fold larger (Fig. 8b), and butaprost was significantly more effective than formoterol (Fig. 8b). However, the difference between formoterol and butaprost vanished with longer exposure times, as the effect of butaprost diminished (Fig. 8c, d). A significant increase in cellular cAMP was also induced by cholera toxin, but the rise occurred with some delay; elevated cellular cAMP levels were observed after 1 and 4 h, but not after 10 min. Surprisingly, IBMX failed to induce a significant rise in cellular cAMP at all time points studied. There was a tendency for a small increase after 4 h, which however failed to be statistically significant. Exposure for 1 or 2 h to cycloheximide (30 μM), which had caused a marked upregulation of β_2_-adrenoceptor mRNA within 1.5 h (Fig. 1), did not cause any increase in cellular cAMP (data not shown, each *n* = 8).

## Discussion

As outlined in the Introduction, due to their bronchodilatatory action, β-adrenoceptor agonists are an essential element in the treatment of chronic obstructive airway diseases. However, other cells, in addition to the airway smooth muscle, might be an addditional target for β-adrenoceptor agonists. Thus, human lung fibroblasts express β_2_-adrenoceptors (Lamyel et al. 2011) which appear to inhibit pro-fibrotic features, such as myo-fibroblast differentiation, proliferation, and collagen synthesis (Liu et al. 2004; Lamyel et al. 2011). Since in chronic obstructive airway disease, β-agonists are applied as long-term treatment, the present study aimed to explore possible agonist-induced changes in β_2_-adrenoceptor expression in human lung fibroblasts.

Previous studies from our laboratory demonstrated that MRC-5 and primary human lung fibroblasts showed very much the same results with regard to the expression and functional response of several G-protein-coupled receptors (Matthiesen et al. 2006; Haag et al., 2008a, b; Ahmedat et al. 2010) and so far studied signal transduction mechanisms (Haag et al. 2008b). Since in particular, MRC-5 and primary human lung fibroblasts showed also the same expression pattern of β-adrenoceptor subtypes, namely a selective expression of β_2_-adrenoceptors, and the same functional response upon β-adrenoceptor activation (Lamyel et al. 2011), MRC-5 cells were used in the present study as a cell line which allows to study physiologically relevant functions in human lung fibroblasts.

The expression of β_2_-adrenoceptors in human lung fibroblasts appears to be highly regulated at the transcriptional level. First, the half-life of β_2_-adrenoceptor mRNA is relatively short; β_2_-adrenoceptor mRNA declined with a half-life of 23 min after addition of actinomycin D. Considering that the inhibition of RNA synthesis may occur with a certain delay after addition of actinomycin D to the culture medium, the half-life of β_2_-adrenoceptor mRNA may even be shorter. Therefore, β_2_-adrenoceptor mRNA levels are expected to reflect immediately changes in β_2_-adrenoceptors gene transcription. In fact, significant changes of β_2_-adrenoceptor mRNA levels were observed already 20 min after drug exposure (Fig. 1). However, the stability of β_2_-adrenoceptor mRNA appears to vary very much in a cell specific manner and may in addition vary with the culture conditions. Thus, a relative short half-life of about 45 and 55 min was observed in DDT_1_-MF-2 hamster smooth muscle cells (Collins et al. 1989) and C6 glioma cells (Hosoda et al. 1995; Danner and Lohse 1997), and in agreement to the present observations, these short half-lives were not affected by agonist exposure. In mononuclear leukocytes, a longer half-life of about 2.7 h was observed which was significantly shorted by agonist exposure (Tittelbach et al. 1998). Strikingly, Danner and Lohse (1997) reported that β_2_-adrenoceptor mRNA in DDT_1_-MF-2 hamster smooth muscle cells largely depended on culture conditions. In cells grown in monolayer culture, the half-life was about 12 h, but in cell grown in suspension culture (the conditions also used in the study of Collins et al. (1989)), it was only 2 h, but under both conditions, it was shorted by about 50 % in presence of isoproterenol. Thus, only in cells, in which the stability of β_2_-adrenoceptor mRNA is high, an agonist-induced destabilization may occur.

Strikingly, β-adrenoceptor agonist exposure evoked a marked upregulation of β_2_-adrenoceptor mRNA expression which was very rapid in onset, but transient and followed by a substantial downregulation. Generally, β-adrenoceptors couple to G_s_ and mediate via activation of adenylyl cyclase an increase in cellular cAMP. In the present study, the time-dependent effects of β-adrenoceptor agonists on β_2_-adrenoceptor mRNA expression were mimicked by activation of adenylyl cyclase by either forskolin or cholera toxin indicating that an increase in cAMP is the crucial signal for these effects. A similar transient β_2_-adrenoceptor–cAMP-mediated upregulation of β_2_-adrenoceptor gene expression has also been described by Collins et al. (1989) in DDT_1_MF-2 hamster smooth muscle cells, and evidence for a cAMP responsive element in the β_2_-adrenoceptor gene was presented (Collins et al. 1990). Furthermore, a cAMP-mediated reduction in β_2_-adrenoceptor mRNA was also observed in transfected Chinese hamster fibroblasts expressing human β_2_-adrenoceptors (Bouvier et al. 1989).

Human lung fibroblasts express also EP_2_ prostanoid receptors (Haag et al. 2008b) which are known to couple to adenylyl cyclase. Short-time exposure to the EP_2_ receptor agonist butaprost induced also an upregulation of β_2_-adrenoceptor mRNA, which—like the effect of forskolin—was not additive to the effect of a β-adrenoceptor agonist, indicating that all three stimuli may act via the same pathway, activation of adenylyl cyclase.

Only the stimulatory effect of β_2_-adrenoceptor agonists on β-adrenoceptor gene expression appears to be the result of a direct, cAMP-mediated regulation of the β_2_-adrenoceptor gene. This is because the stimulatory effect of β_2_-adrenoceptor agonists was (1) blocked by actinomycin D indicating that it was caused by increased transcription and (2) did not require de novo protein synthesis as it was also seen in presence of cycloheximide. On the other hand, the inhibitory effect seen after 4 h agonist exposure was not only prevented by cycloheximide but converted into marked upregulation of β_2_-adrenoceptor mRNA. This un-masking action of cycloheximide indicates that the initial, direct stimulatory signal was still operating but was dominantly opposed by newly synthesized inhibitory factors induced following β_2_-adrenoceptor activation via the adenylyl cyclase–cAMP pathway. Interestingly to note, cycloheximide alone caused a rapid and marked increase in β_2_-adrenoceptor mRNA, indicating that basal β_2_-adrenoceptor gene expression in human lung fibroblasts is under inhibitory control of short-living suppressor proteins. Cycloheximide did not affect cellular cAMP levels, excluding that its effects involve activation of adenylyl cyclase. The observation that actinomycin D prevented the cycloheximide-induced increase in β_2_-adrenoceptor mRNA supports the conclusion that this effect is caused by an increased transcription rather than the result of a prolonged stability of the transcript. Whether the β_2_-adrenoceptor-induced cAMP signal augments the action of these suppressors or induces additional inhibitory regulators remains unknown at present, but it will be a challenge for future studies to identify these regulators which could be potential targets for drugs aiming to improve and maintain β_2_-adrenoceptor function during prolonged agonist exposure.

Although direct activation of adenylyl cyclase by forskolin or cholera toxin mimicked both the initial stimulatory and delayed inhibitory effects of β_2_-adrenoceptor agonists, the non-selective phosphodiesterase inhibitor IBMX mimicked only the delayed inhibitory effect. The reason for that appears to be that the spontaneous activity of adenylyl cyclase is very low and IBMX did not cause any increase in cellular cAMP levels for up to 1 h. Even after 4 h exposure, IBMX did not cause a clear, significant increase in cellular cAMP. Thus, it is even questionable, whether the reduction of β_2_-adrenoceptor mRNA after 4 h exposure to IBMX was caused by changes in cellular cAMP.

Cellular cAMP signaling can be transmitted either by the classic effector protein kinase A (PKA) (e.g., Skålhegg and Taskén 2000) or the alternative cAMP effector Epac (exchange protein activated by cAMP) of which two variants, Epac1 and Epac2, have been identified (de Rooij et al. 1998; Kawasakia et al. 1998). In human lung fibroblasts for example, it has been shown that PKA and Epac differentially regulate proliferation and collagen synthesis (Huang et al. 2008; Haag et al. 2008b). In the present experiments, the selective PKA agonist 6-Bnz-cAMP (Bos 2006; Holz et al. 2008) mimicked the stimulatory effect of the β-adrenoceptor agonist, but only in presence of cycloheximide suggesting that PKA has ability to upregulate directly β-adrenoceptor gene expression, but this action is opposed by PKA-induced inhibitory regulators. On the other hand, Epac appears not to play a major role in the regulation of β_2_-adrenoceptor expression as 8-CPT-2′–O-Me-cAMP, a selective Epac activator (Bos 2006; Holz et al. 2008) did not show any significant effect, neither in absence nor presence of cycloheximide. It should be mentioned that in previous experiments using the same cells and the same concentrations of 6-Bnz-cAMP and 8-CPT-2′–O-Me-cAMP, these agonists mediated a marked and selective inhibition of either the synthesis of collagen or cell proliferation, respectively (Haag et al. 2008b).

In conclusion, expression of β_2_-adrenoceptors in human lung fibroblasts is highly regulated at transcriptional level, suggesting that β_2_-adrenoceptor expression may rapidly respond to physiological or pathological changes as well as pharmacological interventions. The β_2_-adrenoceptor gene appears to be under strong inhibitory control of short-living, not yet identified suppressor proteins. Although both, the time-dependent up- and downregulation of the β_2_-adrenoceptor gene expression by β_2_-adrenoceptor activation appears to be mediated via adenylyl cyclase–cAMP signaling, only the stimulatory effect appears to be a direct action on the β_2_-adrenoceptor gene.
